# A Potential Fatty Acid Metabolism-Related Gene Signature for Prognosis in Clear Cell Renal Cell Carcinoma

**DOI:** 10.3390/cancers14194943

**Published:** 2022-10-09

**Authors:** He Zhang, Di Zhang, Xiaopeng Hu

**Affiliations:** 1 Department of Urology, Beijing Chao-Yang Hospital, Capital Medical University, Beijing 100020, China; 2 Institute of Urology, Capital Medical University, Beijing 100020, China

**Keywords:** clear cell renal cell carcinoma, fatty acid metabolism, gene signature, prognosis, nomogram

## Abstract

**Simple Summary:**

Clear Cell Renal Cell Carcinoma (ccRCC) is the most common and aggressive subtype of renal cancer. Abnormal fatty acid metabolism (FAM) is reported to be strongly associated with multiple malignancies, yet there is limited research in ccRCC. In this manuscript, we reported the significant role of abnormal FAM in predicting the prognosis of ccRCC. Three independent clinical cohorts (TCGA, EMTAB and our clinical cohorts with prognostic profiles and gene expression data, including RNA-seq, microarray and RT-qPCR) were applied as training and two external validation cohorts. As a result, we successfully constructed and validated a novel FAM-related gene signature (FAMGS) and nomogram for the overall survival of patients with ccRCC. Additionally, our study further elucidated the potential immune relevance and molecular mechanisms of abnormal FAM and the signature. In conclusion, the novel FAMGS constructed in this study offered a promising prognostic tool in clinic and potential therapeutic targets for ccRCC patients.

**Abstract:**

This study aims to explore the role of abnormal fatty acid metabolism (FAM) in ccRCC and construct a novel fatty acid metabolism-related gene signature (FAMGS) for prognosis. Three independent ccRCC cohorts, including The Cancer Genome Atlas, E-MTAB-1980 and our clinical cohort (including RNA-seq, microarray and RT-qPCR data), were applied as training and two independent validation cohorts. Firstly, FAM levels were found to be significantly decreased in ccRCC and correlated with degrees of malignancy, confirming the pivotal role of FAM in ccRCC. Applying the least absolute shrinkage and selection operator cox regression, we established a novel FAMGS for overall survival (OS). The FAMGS divided patients into low or high-risk groups in the training cohort and were successfully validated in both the EMTAB and our clinical validation cohorts. Additionally, the FAMGS serves as an independent risk factor for OS of ccRCC. Results of the immune cell abundance identifier (ImmuCellAI) algorithm and gene set variation analysis (GSVA) revealed that patients in the high-risk group have comprehensively impaired metabolism, including lipids, amino acids and tricarboxylic acid cycle-related pathways and a more immunosuppressive tumor microenvironment. In conclusion, our study constructed and validated a novel FAMGS, which may improve the risk stratification optimization and personalized management of ccRCC.

## 1. Introduction

Renal cell carcinoma (RCC) is the second-leading cause of death in urological tumors, with approximately 5% of all new cases among adult men and 3% of all new cases among adult women annually [1]. Clear Cell Renal Cell Carcinoma (ccRCC) is the most prevalent and aggressive subtype of RCC and is responsible for the majority of kidney-cancer-related deaths [2]. Unfortunately, in the absence of useful biomarkers, ccRCC has a poor prognosis at an advanced stage. Therefore, accurate and early prognostic biomarkers are essential for ccRCC patients and in great need in clinical practice.

Fatty acids are required for cell membrane formation, energy storage, signaling molecule production and further biological processes. Increased fatty acid metabolism (FAM) could be a response to the high metabolic demand of cancer cells, which is closely connected to multiple cellular processes, for example, cell growth, cell transformation, tumor development and disease progression [3]. Energy metabolism reprogramming, which can sustain the production of ATP and macromolecules needed for cell growth, division and survival, is a common feature in cancer cells [4]. In addition to glucose metabolism, dysregulation of FAM has also become better understood and appreciated in recent years [5]. An increase in lipid storage was found in the tumor tissues of patients with ccRCC compared with normal kidney tissues. Horiguchi et al. found that fatty acid synthase levels are correlated with tumor aggressiveness and poor patient survival in RCC [6]. Wettersten et al. found that levels of enzymes involved in β-oxidation, the critical step in fatty acid metabolism, decreased in ccRCC tissues [7]. However, the regulatory mechanism of the FAM pathway in ccRCC has not been elucidated. Consequently, identifying fatty acid metabolism-related gene signatures (FAMGS) might open up new avenues for exploring the pathology and treatment of ccRCC.

Our study elucidated the role of FAM in patients with ccRCC and combined different methods to establish the FAMGS for prognosis. Importantly, we successfully validated the prognostic value of the FAMGS in an independent ccRCC cohort and our clinical cohort. Finally, using the FAMGS and clinicopathological features, a nomogram was constructed and verified to enhance prediction power.

## 2. Materials and Methods

### 2.1. Data source and Processing

The RNA-sequencing data of 530 ccRCC patients with available follow-up data obtained from the TCGA database [8] was used as the training cohort. E-MTAB-1980 (EMTAB) cohort (with 101 samples with expression profiles) obtained from the ArrayExpress database [9] served as validation cohorts. In our clinical cohort, the following criteria were used to select 21 pairs of ccRCC and adjacent normal samples: (1) patients with surgical treatment at the Beijing Chao-Yang Hospital; (2) patients without any neoadjuvant chemotherapy and other special treatments; (3) patients who underwent initial surgery in our hospital. Two pathologists identified the final diagnosis of the samples. Additionally, the microarray dataset GSE73731 accessed from GEO [10] served as another independent cohort for the subgroup analysis. The clinical characteristics of the TCGA, EMTAB and our clinical cohorts are shown in Table 1. This study was carried out in accordance with The Code of Ethics of the World Medical Association (Declaration of Helsinki). All patients signed the informed consent and this study was approved by the ethics committees of Beijing Chao-Yang Hospital.

### 2.2. Candidate Selection and Signature Establishment

We extracted the FAM-related gene set (HALLMARK_FATTY_ACID_METABOLISM) which contains 158 genes from Molecular Signatures Database v7.4 [11]. Single-sample gene set enrichment analysis (ssGSEA) was employed to calculate the performance of the FAM-related gene set in the training cohort. Univariate Cox proportional hazard regression analysis was applied to recognize prognostic genes with a *p*-value < 0.05 in the training cohort. Then least absolute shrinkage and selection operator (LASSO) Cox regression analysis was used to further establish the optimal FAM gene signature [12]. The risk score formula of the signature is derived by using the LASSO regression coefficient and normalized expression level of genes: the FAMGS risk score = $\sum_{i}^{n}\mathrm{Coefficient}\left({\mathrm{mRNA}}_{i}\right)\times \;\mathrm{Expression}\left({\mathrm{mRNA}}_{i}\right)$.

### 2.3. Quantitative RT-qPCR and Risk Score Calculations of Clinical Cohort

The HiScript III RT SuperMix Kit (R32301, Vazyme, Nanjing, China) was used to reverse transcribe total RNA from clinical samples into cDNA. The expression levels of RNAs were determined using the AceQ qPCR SYBR Green Master Mix (R323-01, Vazyme, China) by ABI 7500 (Applied Biosystems, Foster City, CA, USA). Based on the 2−ΔΔCt method, expression levels of target RNAs were normalized to GAPDH. Table S1 lists the used primers. Risk scores for each ccRCC patient in the clinical cohort were calculated according to the previous studies using non-normalized cycle threshold (Ct) values based on the formula: mean Ct (ACAA2, ACADL, ALDH3A2, CPT2, HGPD, PTPRG)—mean Ct (CPOX, ENO2, ENO3, TDO2) [13,14].

### 2.4. Bioinformatics and Statistical Analyses

R software (version 4.1.2) was used to process data. We divided patients in the TCGA training cohort into low and high-risk groups according to the optimal cutoff risk score measured by the R package “survival” [15] and the remaining validation cohorts were assigned by the group proportion of the training cohort. Univariate and multivariate cox analysis was conducted with the R package “survival”. The R package “survival ROC” was used to evaluate the prognostic role of Kaplan–Meier (K-M) curves and time-dependent receiver operating characteristic (tROC) [16]. LASSO analysis was conducted using the R package “glmnet” [17]. Additionally, stratification analysis was conducted based on the clinical characteristics of different subgroups. An annotated gene set file (msigdb.v7.4.entrez.gmt) was selected to perform GSVA, which used the FAMGS risk scores to explore potential biological functions [18]. A web tool Immune cell abundance identifier (ImmuCellAI) was used to analyze the tumor immune status of each patient [19]. The R “pheatmap” package was used to generate a heatmap plot [20]. The R package ‘rms’ was applied to plot nomograms and calibration curves [21]. Decision curve analysis (DCA) was conducted to measure the clinical benefits with the R package ‘ggDCA’. Categorical variables were presented as counts (percentages), while continuous variables were presented as median with interquartile range (IQR). Kruskal−Wallis test was used to analyze differences between multiple groups and Wilcoxon test was used to compare differences between two groups. This study considered the *p*-value < 0.05 as statistically significant.

## 3. Results

### 3.1. Study Design and Cohort Characteristics

**Figure 1:**
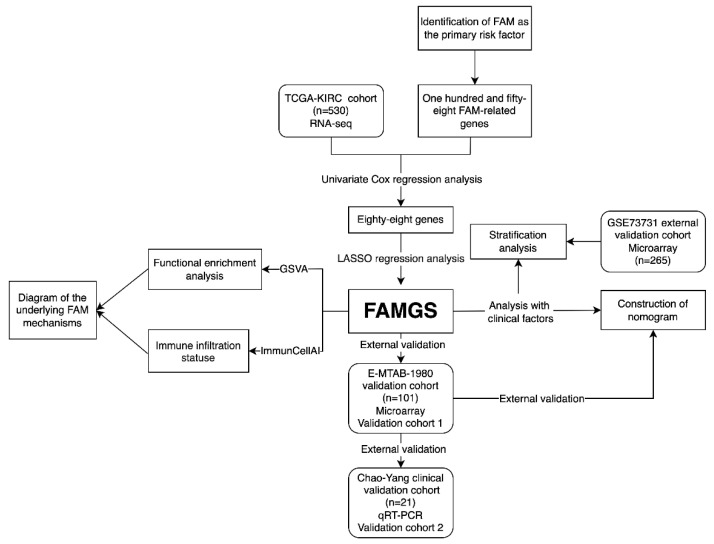
Flow diagram of study design. FAM: Fatty acid metabolism; TCGA: The Cancer Genome Atlas; KIRC: Kidney renal clear cell carcinoma; LASSO: Least absolute shrinkage and selection operator; FAMGS: Fatty acid metabolism gene signature; GSVA: Gene set variation analysis; ImmunCellAI: Immune cell abundance identifier; qRT-PCR: Real-time Quantitative Polymerase Chain Reaction.

In the beginning, we confirmed the hypothesis that the FAM served as the major risk factor for OS in ccRCC patients. Next, Univariate Cox and LASSO Cox regression were combined to establish the FAMGS for overall survival. Subsequently, we evaluated the prognostic value of the FAMGS in the validation cohort. Additionally, stratification analysis of clinical characteristics was performed in the training cohort and two independent validation cohorts. Functional enrichment and immune cell analysis were employed to further explore the signature’s potential molecular mechanisms and immune relevance. Subsequently, we summarized a speculative mechanism diagram. Finally, we construct and validate the nomogram based on the FAMGS in the TCGA training and EMTAB, respectively. A flow diagram is shown in Figure 1.

Clinical baseline characteristics of patients in the TCGA training cohort, EMTAB validation cohort and Chao-Yang clinical validation cohort are described in Table 1. The median (IQR) OS in the TCGA training cohort was 1181 (520–1912) days, which had 357 (67.36%) deaths. The E-MTAB-1980 validation cohort contained 101 patients, who had a median (IQR) OS of 1530 (1020–2430) days and 23 (22.77%) deaths. In the Chao-Yang validation cohort, the median (IQR) OS was 848 (712–916) days, with 18 (85.71%) deaths. Additionally, we collected clinicopathological data for all patients, which included age, gender, tumor grade and AJCC stage.

### 3.2. Fatty Acid Metabolism Confirmed as a Crucial Factor in ccRCC

**Figure 2:**
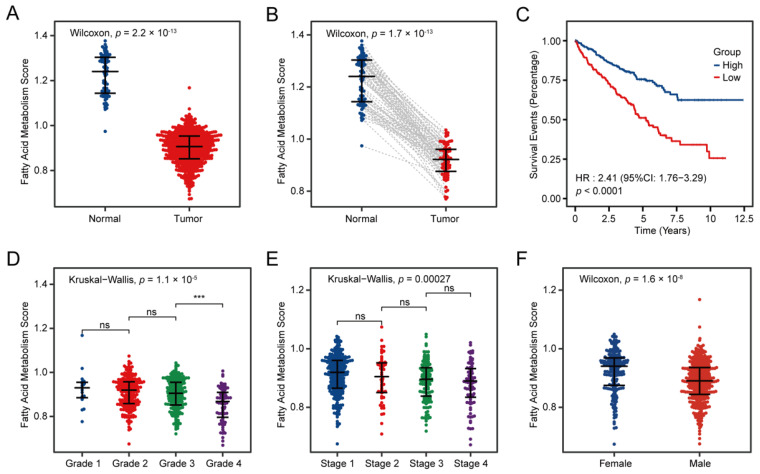
Clinical correlation of fatty acid metabolism pathway in patients with ccRCC. Beeswarm plots comparing the ssGSEA scores in all ccRCC samples and all adjacent normal tissues (**A**), paired ccRCC samples and adjacent normal tissues (**B**) and different tumor grades (**D**), AJCC stage (**E**) and gender (**F**). (**C**) Kaplan–Meier analysis showed that patients with lower ssGSEA scores of fatty acid metabolism exhibited worse OS. ccRCC: Clear Cell Renal Cell Carcinoma; ssGSEA: Single-sample gene set enrichment analysis; AJCC: American Joint Committee on Cancer; HR: Hazard ratio; CI: Confidence interval. *p* values less than 0.05 were considered to be statistically significant. ns: No significance, *** *p* < 0.001.

The ssGSEA enrichment score of the FAM pathway was calculated for 530 cancer tissues and 72 cancer-adjacent normal tissues in the TCGA training cohort. We observed a lower FAM enrichment score in both paired and non-paired tumors compared with normal kidney tissues (*p* < 0.0001; Figure 2A,B). Then, we used the median cutoff of the ssGSEA enrichment score to subdivide tumor samples into two groups. The beeswarm plots indicated that patients with different clinical characteristics, including gender, tumor grade, AJCC stage and status, had strikingly different ssGSEA enrichment scores (*p* < 0.001; Figure 2D–F), but they were not substantially different with different ages (*p* = 0.33; Figure S1A). More remarkably, The K–M survival analysis showed that the lower FAM enrichment score has a pooled hazard ratio (HR; 95% confidence interval (CI)) = 2.41 (1.76–3.29) and *p* < 0.0001 (Figure 2C).

### 3.3. Construction and Validation of the FAMGS for Prognosis

According to the univariate Cox analysis, 88 candidate prognostic FAM-related genes were identified (*p* < 0.05; Table S2). Afterward, Univariate Cox proportional hazard regression analysis was employed to screen prognostic genes for ccRCC. According to the results of ten-fold cross-validation, we chose the model of λ value of 0.0501 to overcome overfitting (Figure S1B,C). Finally, the LASSO Cox algorithm was used to integrate 10 genes to establish the FAMGS. Each patient’s risk score is calculated as follows: risk score = (−0.2219 × expression level of CPT2) + (−0.0416 × expression level of ACADL) + (−0.2152 × expression level of PTPRG) + (0.0897 × expression level of CPOX) + (0.0429 × expression level of *TDO2) + (−0.0428 × expression level of *HPGD) + (0.0104 × expression level of *ENO2) + (0.0944 × expression level of *ENO3) + (−0.0298 × expression level of *ALDH3A2) + (−0.0536 × expression level of *ACAA2). LASSO coefficients for FAMGS are shown in Figure S1D. Following the optimal cutoff value calculated from the FAMGS risk scores, patients were categorized into low- and high-risk groups.

**Figure 3:**
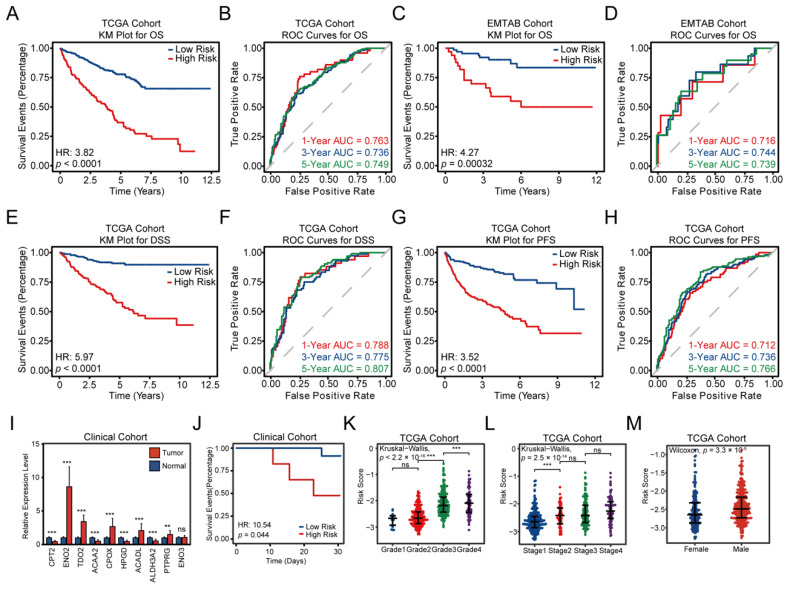
Construction and validation of the fatty acid metabolism-related prognostic signature. Kaplan–Meier survival curves of OS (**A**), DSS (**E**) and PFS (**G**) in the TCGA cohort. 1- (**B**), 3- (**F**) and 5- (**H**) year-dependent ROC curves of OS, DSS and PFS in the TCGA cohort. Kaplan–Meier survival curves of OS in the EMTAB (**C**) and clinical cohort (**J**). (**D**) 1-, 3- and 5-year-dependent ROC curves of OS in EMTAB cohort. (**I**) The barplot exhibits the expressions of ten genes in FAMGS evaluated by RT-qPCR in 21 ccRCC samples and paired adjacent normal samples. Beeswarm plots comparing the FAMGS risk score in different tumor grades (**K**), AJCC stage (**L**) and gender (**M**). OS: Overall survival; DSS: Disease-specific survival; PFS: Progression-free survival; ccRCC: Clear Cell Renal Cell Carcinoma; TCGA: The Cancer Genome Atlas; EMTAB: E-MTAB-1980; ROC: Receiver operating characteristic; FAMGS: Fatty acid metabolism gene signature; RT-qPCR: Real-time Quantitative Polymerase Chain Reaction; HR: Hazard ratio; CI: Confidence interval. *p* values less than 0.05 were considered to be statistically significant. ns: No significance, ** *p* < 0.01, *** *p* < 0.001.

The K–M survival plots demonstrated that patients with higher FAMGS risk scores exhibited worse OS, DSS and PFS in the TCGA training cohort (HR = 3.82, 95% CI = 2.82–5.18, *p* < 0.0001; HR = 5.97, 95% CI = 3.63–9.81, *p* < 0.0001; HR = 3.52, 95% CI = 2.48–4.99, *p* < 0.0001; Figure 3A,E,G). Then, the prognostic value of FAMGS for OS was validated in the EMTAB validation cohorts (HR = 4.27, 95% CI = 1.81–10.07, *p* = 0.00032; Figure 3C) and Chao-Yang clinical cohort (HR = 10.54, 95% CI = 1.067–104.2, *p* = 0.044; Figure 3J). To investigate differential expression in tumor tissues and normal tissues, real-time quantitative polymerase chain reaction (RT-qPCR) was used to analyze the expression of the ten FAMGS genes between 21 pairs of ccRCC and adjacent normal samples. Clinical samples showed significant downregulation of the following genes: CPT2, ACAA2, HPGD and ALDH3A2, while the expression of ENO2, TDO2, CPOX, ACADL and PTPRG was significantly upregulated in the tumor tissue (*p* < 0.01; Figure 3I). The tROC analysis further validated the prognostic value of the FAMGS (Figure 3B,F,H). The areas under the curve (AUCs) of the FAMGS were evaluated and then results showed 1-year (AUC = 0.763), 3-year (AUC = 0.736) and 5-year (AUC = 0.749) OS, 1-year (AUC = 0.788), 3-year (AUC = 0.775) and 5-year (AUC = 0.807) DSS and 1-year (AUC = 0.712), 3-year (AUC = 0.736) and 5-year (AUC = 0.766) PFS in the TCGA training cohort. Moreover, we verified the AUCs or 1-year (AUC = 0.716), 3-year (AUC = 0.744) and 5-year (AUC = 0.739) OS in the EMTAB cohort(Figure 3D). The above results all demonstrated the high-prediction accuracy of our signature, while the K–M survival analysis (HR = 1.09, 95% CI = 0.39–3.07, *p* = 0.87; Figure S1E) and tROC analysis (1-year AUC = 0.571, 3-year AUC = 0.511, 5-year AUC = 0.505; Figure S1F) of DFI in the TCGA training cohort showed no difference. To determine the prognostic value of FAMGS, a stratification analysis was conducted. According to four clinical characteristics (age, sex, tumor grade and AJCC stage), patients in the TCGA cohort, EMTAB cohort and GSE73731 cohort were divided into two groups. Among three cohorts, the FAMGS maintained a highly consistent predictive capacity for ccRCC patients in subgroups of tumor grade, AJCC stage and gender (Figure 3K–M and Figure S2B–D,F–H). Meanwhile, the results of the age subgroup did not show a significant difference in the TCGA cohort (*p* = 0.27; Figure S2A) and EMTAB cohort (*p* = 0.7; Figure S2E).

### 3.4. Comprehensive Enrichment Analyses and Immune Infiltration

**Figure 4:**
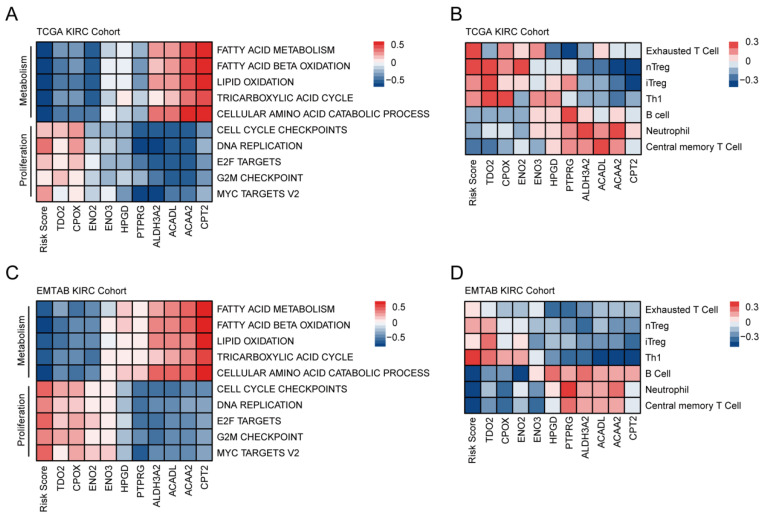
Results of GSVA and ImmuCellAI. Heatmap for correlations between GSVA scores, FAMGS risk scores and gene expressions in the TCGA training cohort (**A**) and EMTAB validation cohort (**C**). Heatmap for correlation between infiltration scores, FAMGS risk scores and gene expressions in the TCGA training cohort (**B**) and EMTAB validation cohort (**D**). GSVA: Gene set variation analysis; ImmuCellAI: Immune cell abundance identifier; FAMGS: Fatty acid metabolism gene signature; TCGA: The Cancer Genome Atlas; EMTAB: E-MTAB-1980.

Firstly, GSVA was conducted to better understand biological processes that may affect the prognosis. Correlation analyses were performed between GSVA scores of each pathway and genes of the FAMGS in the TCGA training cohort and EMTAB validation cohort (Table S3). A high level of FAMGS risk scores and risk gene expressions significantly correlated with metabolism inhibition and cell proliferation (Figure 4A), which indicated a poor prognosis in high-risk patients. Additionally, in the validation set, similar results were investigated (Figure 4C).

The infiltration levels of 24 immune cells were obtained from the ImmuCellAI and then correlation analysis was performed for utilizing infiltration scores, risk scores and gene expressions. The results of the TCGA training and the EMTAB validation datasets both showed that risk scores had a significantly positive correlation with regulatory T cells and exhausted T cells, while being inversely correlated with major immune effector cells, including B cells, neutrophils and central memory T Cells (Table S4; Figure 4B,D). Based on the observations above, high-risk scores indicated the immunosuppressive status of the tumor environment.

**Figure 5:**
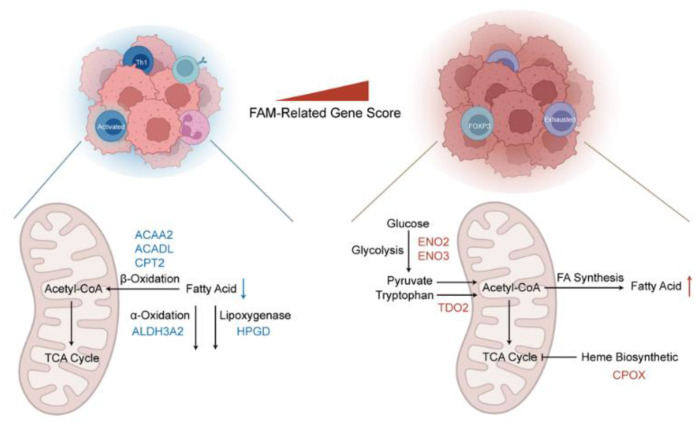
Diagram of the underlying FAM mechanisms for ccRCC. FAM: Fatty acid metabolism; ccRCC: Clear Cell Renal Cell Carcinoma. The blue arrow represents the decrease of fatty acid synthesis, and the red arrow represents the increase in fatty acid synthesis. The black arrow represents the pathway of fatty acid synthesis and decomposition.

Figure 5 summarizes and demonstrates a speculative mechanism diagram containing FAM-related aberrant metabolic alterations and immune cell clustering. Risk genes, including ENO2, ENO3, TDO2 and CPOX, have high expression levels in the high FAM-related gene score groups and promoted glycolysis, acetyl-CoA synthesis and heme biosynthetic, respectively, which led to an increase in fatty acid synthesis and immunosuppressive microenvironment in tumor tissue. In contrast, protective genes, including ACAA2, ACADL, CPT2, ALDH3A2 and HPGD, have high expression levels in the low FAM-related gene score group. The decrease in fatty acid is due to an increased degradation in the a-oxidation, B-oxidation and Lipoxygenase pathways.

### 3.5. Establishment and Verification of a Nomogram Model According to the FAMGS

**Figure 6:**
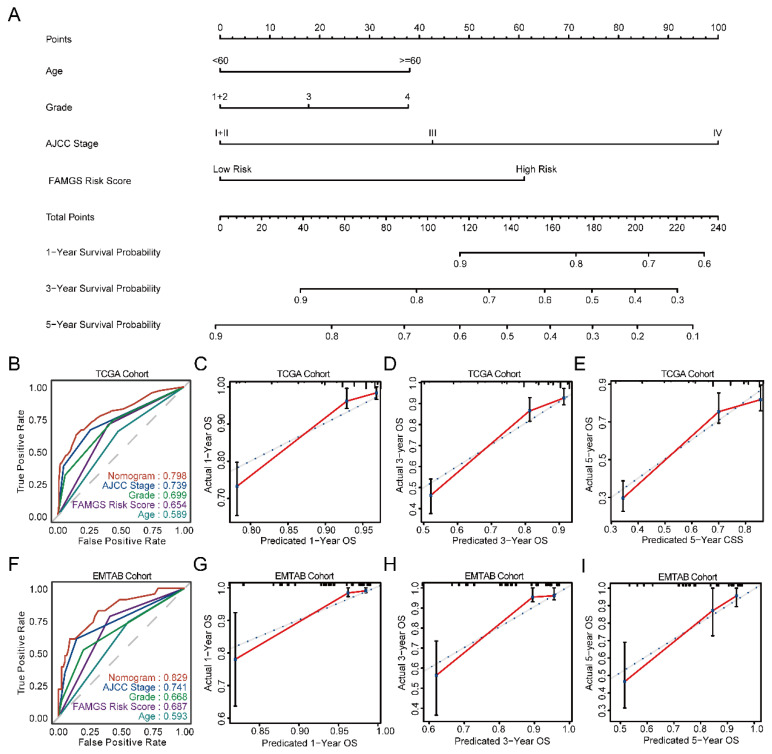
Construction and validation of a nomogram of OS for ccRCC patients. (**A**) Nomogram for predicting the probability of 1-, 3- and 5-year OS for ccRCC patients. Time-dependent ROC curves of the nomogram, the FAMGS, age, tumor grade and AJCC stage in the TCGA training cohort (**B**) and EMTAB validation cohort (**F**). Calibration plots of the nomogram for predicting the probability of OS at 1, 3 and 5 years in the TCGA training cohort (**C**–**E**) and EMTAB validation cohort (**G**–**I**). OS: Overall survival; ROC: Receiver operating characteristic; FAMGS: Fatty acid metabolism gene signature; AJCC: American Joint Committee on Cancer.

To investigate prognostic factors for OS in TCGA cohorts, univariate and multivariate Cox regression analyses were conducted in the TCGA training cohort. As a result, the FAMGS and three clinical factors (including age, AJCC stage and tumor grade) were screened out to be independent predictors of prognosis (*p* < 0.05) for ccRCC (Table 2). By integrating independent clinical prognostic factors, we constructed a nomogram allowing ccRCC patients to assess 1-, 3- and 5-year OS (Figure 6A). As demonstrated by receiver operating characteristic (ROC) curves, the nomogram was more accurate in predicting than either single factor (Figure 6B). After that, tROC of 1-, 3- and 5-year OS also indicated an improved prediction (Figure S3A–C). Moreover, the calibration plots for one, three and five years of OS demonstrated excellent consistency between observations and predictions (Figure 6C–E). Finally, DCA curves suggested that our nomogram has wide clinical practicability (Figure S3G–I).

Then, the EMTAB cohort was utilized to verify the predictive ability of the nomogram. Univariate and multivariate Cox regression analyses were carried out to validate the FAMGS’s predictive power. The results showed that the signature could independently predict the prognosis of patients with ccRCC. Based on the fixed nomogram formula of the training cohort, we calculated scores of the signature and each prognostic predictor. The ROC revealed the developed model retains good predictive performance (Figure 6F). In addition, a good agreement is found in the results of 1-, 3- and 5-year calibration curves for OS (Figure 6G–I). Furthermore, tROC of 1-, 3- and 5-year OS (Figure S3D–F) and DCA curves of predicted 1-, 3- and 5-year OS (Figure S3J–L) indicated the nomogram model showed a better performance than other independent predictors.

## 4. Discussion

ccRCC is one of the most common subtypes of RCC, with high mortality and unsatisfied prognosis [2]. Radiation tests and renal biopsy are the most common methods of diagnosing and surgical resection is the main therapeutic method for ccRCC patients [22]. However, the curative effect and prognosis of terminal ccRCC patients are poor. A deeper understanding of molecular mechanisms and biomarkers is urgent to explore individualized treatment improvements and prognostic evaluation. In ccRCC, increased lipid uptake, storage and lipogenesis contribute to rapid tumor growth via metabolic reprogramming [23]. As a metabolic disease, ccRCC is generally associated with reprogramming of the tricarboxylic acid cycle, glucose metabolism and fatty acid metabolism [7]. Due to intensive metabolic reprogramming studies, the role of fatty acid metabolism in malignancies has been increasingly recognized over recent years [4,24,25,26]. To date, there have been several fatty acid metabolism gene signatures developed for different types of cancer, such as hepatocellular carcinoma [27], colorectal cancer [28], glioma [29] and ccRCC [30]. However, the signature in ccRCC lacks effective performance evaluation, such as the concordance index or the AUC and external validation of the prognostic value. Although some evidence from previous studies demonstrated increased lipid storage and utilization of lipids for membrane synthesis in ccRCC, the specific role of FAM in terms of prognosis or functional contribution in ccRCC still needs further study and confirmation [2,7,31]. In order to address the above problems, our study aims to apply multiple cohorts to investigate and verify the prognostic value of FAM in ccRCC.

This study confirmed FAM as an important predictor of survival among ccRCC patients. We establish a FAMGS of great prognostic value for OS utilizing TCGA RNA-sequencing data. The FAMGS prognostic value was then verified via microarray and qRT-PCR data, including an independent cohort and our clinical cohort, which showed consistent and significant results. The three datasets used as training and validation cohorts were based on three different platforms and methods (TCGA: RNA-seq, E-MTAB-1980: microarray and Chaoyang clinical cohort: qRT-PCR), which robustly confirmed the reliability of our novel gene signature and indicated a strong translational potential. When used across different platforms in sub-group analysis, the FAMGS still showed a significant ability to discriminate against high-risk patients, indicating that it can be used in pooled populations. Moreover, after integrating traditional clinical characteristics in ccRCC patients, FAMGS remained an independent prognostic factor in both the training and validation cohorts, processing superior prognostic value to the clinical characteristics, including tumor grade and age. The FAMGS and other clinical characteristics were integrated into a nomogram model to develop predictive ability. The nomogram has proven to be an accurate prognostic indicator for ccRCC patients based on ROC curve analysis and DCA curves. In addition, a good calibration of the OS nomogram was observed in the validation set.

The ccRCC patients had higher levels of fatty acylcarnitines and carnitine than normal controls; nevertheless, the specific mechanisms of different lipid metabolism, including fatty acid metabolism, have not yet been systematically investigated [7]. According to the robust results, we summarized a speculative mechanism diagram combining potential molecular mechanisms and immune relevance of the FAMGS. The results revealed that tumor tissue with high FMAGS had metabolic disorders, including FAM, amino acid metabolism, heme metabolism and glucose metabolism. We found the increased expression of glycolysis-related genes (ENO2 and ENO3), heme biosynthetic-related genes (CPOX) and amino acid catabolism-related genes (TOD2) all contributed to the increased fatty acid production. In contrast, expression of normal lipid catabolism pathway-related genes, including α-Oxidation gene (ALDH3A2), β-Oxidation genes (ACAA2, ACADL and CPT2) and lipoxygenase-related gene (HPGD), was found to increase, which led to a reduction in fatty acid accumulation. Previous studies that showed metabolic alterations modify the tumor microenvironment meet our results [32]. At the single-cell level, the FAM hallmark pathway of ccRCC was found significantly repressed compared with normal renal tubular epithelium [33]. We found accumulation of Exhausted T cells, Natural and Induced T Regulatory Cells in the tumor tissue with high FAMGS. It is worth noting that Lim et al. found that lipid metabolism, especially fatty acid, is one of the most enriched pathways in intratumoral Treg cells [34]. In the TME, with increasing FAMGS in the tumor tissue, immune cells undergo a switch from an immune-active to an immune-suppressive state. Above all, this contributes to the poor prognostic outcome for the patients. Moreover, the diagram contains information about the sites of action of relevant biomarkers in fatty acid metabolism. The detailed mechanism of some biomarkers was investigated in other cancer types. For example, CPT2, a protective biomarker in our study, was found in E2F1 and E2F2-mediated repression of CPT2, providing a lipid-rich environment required for hepatocarcinogenesis [35]. ENO3 inhibits the growth and metastasis of HCC through Wnt/β-Catenin signaling pathway but serves as a risk biomarker in our model [36]. In addition, TDO2 has the function of transducing the tumor immune escape via the IDO1/TDO2–KYN–AhR signaling pathway [37]. The FAMGS contains five biomarkers that were studied in ccRCC, but others have never been examined [38,39,40,41]. In summary, further investigation of the biological functions associated with the FAMGS is required in ccRCC.

However, our research still has some limitations. Firstly, as this is a retrospective study, the FAMGS needs to be further validated in prospective trials to confirm its prognostic robustness and clinical usefulness. Secondly, it is necessary to carry out more experiments to clarify the biological functions of fatty acid metabolism underlying the FAMGS.

## 5. Conclusions

In conclusion, we established and verified the FAMGS and nomograms for prognosis in ccRCC, which could improve risk stratification and guide FAM-targeted treatment.

## Figures and Tables

**Table 1:** Clinical characteristics of patients in the TCGA training, EMTAB validation cohorts and Chao-Yang clinical validation cohort.

Characteristics		TCGA Training Cohort	EMTAB Validation Cohort	Chao-Yang Validation Cohort
Number of Patients		530	101	21
Overall Survival (IQR)		1181.5 (520, 1912)	1530 (1020, 2430)	848 (712,916)
Overall Survival Status (%)	Survival	357 (67.36)	78 (77.23)	17 (80.95%)
	Deceased	173 (32.64)	23 (22.77)	4 (19.05%)
Age (IQR)		61 (52, 70)	64 (56, 72)	67 (62.5, 72.5)
Gender (%)	Male	344 (64.91)	77 (76.24)	16 (76.19%)
	Female	186 (35.09)	24 (23.76)	5 (23.81%)
Grade (%)	G1	14 (2.64)	13 (12.87)	2 (9.52%)
	G2	227 (42.83)	59 (58.42)	14 (57.14%)
	G3	207 (39.06)	22 (21.78)	5 (23.81%)
	G4	74 (13.96)	5 (4.95)	0 (0.00)
	Not Available	8 (1.51)	2 (1.98)	0 (0.00)
AJCC Stage (%)	Stage I	265 (50.00)	66 (65.35)	13 (61.91%)
	Stage II	57 (10.75)	10 (9.90)	4 (19.05%)
	Stage III	123 (23.21)	13 (12.87)	2 (9.52%)
	Stage IV	82 (15.47)	12 (11.88)	2 (9.52%)
	Not Available	3 (0.57)	0 (0.00)	0 (0.00)

Abbreviations: TCGA: The Cancer Genome Atlas; EMTAB: E-MTAB-1980; IQR: Interquartile range; AJCC: American Joint Committee on Cancer.

**Table 2:** Univariate and multivariate Cox regression analyses for predicting OS in the TCGA training cohort and EMTAB validation cohort.

TCGA Training Cohort						
	Univariate			Multivariate		
Factors		HR (95% CI)	*p* Value		HR (95% CI)	*p* Value
FAMGS Risk Score		3.729 (2.752–5.053)	<0.001		2.647 (1.911–3.673)	<0.001
Age		1.825 (1.333–2.5)	<0.001		1.624 (1.18–2.234)	0.003
Gender		0.941 (0.691–1.283)	0.7			
Grade	G1 + G2	1		G1 + G2	1	
	G3	1.947 (1.339–2.832)	<0.001	G3	1.222 (0.823–1.813)	0.321
	G4	5.235 (3.521–7.787)	<0.001	G4	1.616 (1.01–2.587)	0.045
AJCC Stage	Stage I + II	1		Stage I + II	1	
	Stage III	2.51 (1.713–3.678)	<0.001	Stage III	1.75 (1.172–2.611)	0.006
	Stage IV	6.192 (4.341–8.833)	<0.001	Stage IV	3.618 (2.403–5.448)	<0.001
EMTAB Validation Cohort						
	Univariate			Multivariate		
Factors		HR (95% CI)	*p* value		HR (95% CI)	*p* value
FAMGS Risk Score		4.419 (1.872–10.431)	<0.001		2.964 (1.073–8.184)	0.036
Age		2.262 (0.891–5.747)	0.086		1.717 (0.604–4.823)	0.313
Gender		0.441 (0.131–1.486)	0.187			
Grade	G1 + G2	1		G1 + G2	1	
	G3	3.015 (1.247–7.288)	0.014	G3	1.193 (0.425–3.347)	0.738
	G4	12.378 (3.222–47.557)	<0.001	G4	3.277 (0.699–15.351)	0.132
AJCC Stage	Stage I + II	1		Stage I + II	1	
	Stage III	5.651 (1.985–16.081)	0.001	Stage III	3.284 (1.084–9.948)	0.036
	Stage IV	9.298 (3.551–24.341)	<0.001	Stage IV	6.246 (2.116–18.438)	<0.001

Abbreviations: OS: Overall survival; TCGA: The Cancer Genome Atlas; EMTAB: E-MTAB-1980; HR: Hazard ratio; CI: Confidence interval. FAMGS: Fatty acid metabolism gene signature; AJCC: American Joint Committee on Cancer. *p* values less than 0.05 were considered to be statistically significant.

## Data Availability

All data generated or analyzed during this study are included in the published article and its supplementary materials files.

## Supplementary Materials

The following are available online at https://www.mdpi.com/article/10.3390/cancers14194943/s1, Table S1: Sequences of primers used for real-time quantitative polymerase chain reaction. Table S2: Identified 88 candidate genes by Univariate Cox regression analyses for predicting OS in the TCGA training cohort. Table S3: Correlation analysis with GSVA scores, FAMGS risk scores and gene expressions in the TCGA training cohort and EMTAB validation cohort. Table S4: Correlation analysis with ImmuCellAI infiltration scores, FAMGS risk scores and gene expressions in the TCGA training cohort and EMTAB validation cohort. Figure S1: (A) Beeswarm plots comparing the ssGSEA scores in age. (B,C) The LASSO Cox regression model was used to identify the most robust markers, with an optimal\lambda value of 0.0617. (D) Distribution of LASSO coefficients of the FAM-related gene signature. (E) Kaplan–Meier survival curves of DFI in TCGA cohort. (F) 1-, 3- and 5-year-dependent ROC curves of DFI in TCGA cohort. ssGSEA: Single-sample gene set enrichment analysis; LASSO: Least absolute shrinkage and selection operator; FAM: Fatty acid metabolism; DFI: Disease-free interval; TCGA: The Cancer Genome Atlas; ROC: Receiver operating characteristic; HR: Hazard ratio; CI: Confidence interval. *p* values less than 0.05 were considered to be statistically significant. Figure S2: Stratification analysis of FAMGS. Beeswarm plots comparing the FAMGS risk score in the age in the TCGA training cohort (A) and EMTAB validation cohort (E). Beeswarm plots comparing the FAMGS risk score in different tumor grades, AJCC stage and gender in the TCGA training cohort (B–D) and EMTAB validation cohort (F–H). FAMGS: Fatty acid metabolism gene signature; TCGA: The Cancer Genome Atlas; EMTAB: E-MTAB-1980; AJCC: American Joint Committee on Cancer. *p* values less than 0.05 were considered to be statistically significant. ns: no significance, * *p* < 0.05, *** *p* < 0.001. Figure S3: ROC and DCA curves. ROC curves of 1-, 3- and 5-year OS predicted by the nomogram in the TCGA training cohort (A–C) and EMTAB validation cohort (D–F). DCA plots of the nomogram, the FAMGS and clinical characteristics for predicting the probability of OS at 1, 3 and 5 years in the TCGA training cohort (G–I) and EMTAB validation cohort (J–L). ROC: Receiver operating characteristic; DCA: Decision curve analysis; TCGA: The Cancer Genome Atlas; EMTAB: E-MTAB-1980; FAMGS: Fatty acid metabolism gene signature; OS: Overall survival.
