# Supplementary material for: A Potential Fatty Acid Metabolism-Related Gene Signature for Prognosis in Clear Cell Renal Cell Carcinoma
Source: Cancers (Basel). 2022 Oct 9;14(19):4943. doi: 10.3390/cancers14194943 (PMC9564311; doi:10.3390/cancers14194943)
Supplement: Supplementary file 1 [file cancers-14-04943-s001.zip › Table S1.pdf]

Supplementary Table S1. Sequences of primers used for quantitative real-time polymerase chain reaction (qRT-PCR)

Gene name	Forward primer	Reverse primer
ACAA2	AGACAATGCAGGTAGACGAGC	ACCCATGATAGAGGGATCACATC
ACADL	TGCAATAGCAATGACAGAGCC	CGCAACTACAATCACAACATCAC
ALDH3A2	AAACCAGTTAAGAAGAACGTGCT	CGAAGGGGTAATTCCAAGCTC
CPOX	TGATGATCTTGACTCTCCGTCC	AGTGCTTTTTCACAAGGGGAA
CPT2	GGGGCCTACCTGGTCAATG	TGGGTAAACGAGTTGAGTTGAAA
ENO2	AGCCTCTACGGGCATCTATGA	TTCTCAGTCCCATCCAACTCC
ENO3	TATCGCAATGGGAAGTACGATCT	AAGCTCTTATACAGCTCTCCGA
HPGD	GCTGGTGGATTGGAATCTTGA	CCAGGGCAGCTTTACACTGT
PTPRG	TGGTGCCTATGGTCCTGAG	CGCGCATACTGGTCTAAAATGTC
TDO2	AAGGTTGTTTCTCGGATGCAC	TGTCATCGTCTCCAGAATGGAA
