# Supplementary material for: A Potential Fatty Acid Metabolism-Related Gene Signature for Prognosis in Clear Cell Renal Cell Carcinoma
Source: Cancers (Basel). 2022 Oct 9;14(19):4943. doi: 10.3390/cancers14194943 (PMC9564311; doi:10.3390/cancers14194943)
Supplement: Supplementary file 1 [file cancers-14-04943-s001.zip › Table S2.pdf]

Supplementary Table S2. Identified 88 candidate genes by Univariate Cox regression analyses for predicting OS in the TCGA training cohort

uni_cox_sig_genes	Hazard Ratio(95% CI)	*p* value
UROD	0.668(0.487_0.916)	0.012
CYP4A11	0.875(0.828_0.924)	$p < 0.001$
CYP4A22	0.818(0.746_0.898)	$p < 0.001$
CPT2	0.507(0.417_0.617)	$p < 0.001$
CRYZ	0.725(0.645_0.815)	$p < 0.001$
ACADM	0.647(0.568_0.737)	$p < 0.001$
HAO2	0.832(0.778_0.889)	$p < 0.001$
HMGCS2	0.849(0.797_0.905)	$p < 0.001$
SDHC	0.642(0.518_0.796)	$p < 0.001$
ALDH9A1	0.677(0.558_0.821)	$p < 0.001$
FMO1	0.858(0.8_0.919)	$p < 0.001$
G0S2	1.202(1.095_1.32)	$p < 0.001$
ODC1	0.811(0.682_0.966)	0.019
HADHB	0.581(0.471_0.717)	$p < 0.001$
RETSAT	0.559(0.46_0.68)	$p < 0.001$
GPD2	0.695(0.569_0.848)	$p < 0.001$
HIBCH	0.52(0.428_0.632)	$p < 0.001$
ACADL	0.732(0.667_0.803)	$p < 0.001$
ACAA1	0.639(0.505_0.81)	$p < 0.001$
PTPRG	0.598(0.513_0.697)	$p < 0.001$
CPOX	1.363(1.036_1.794)	0.027
MGLL	0.754(0.619_0.92)	0.005
EHHADH	0.728(0.662_0.801)	$p < 0.001$
HSD17B11	0.8(0.678_0.945)	0.009
RAP1GDS1	0.537(0.408_0.706)	$p < 0.001$
METAP1	0.566(0.444_0.721)	$p < 0.001$
TDO2	1.286(1.121_1.476)	$p < 0.001$
ETFDH	0.507(0.411_0.626)	$p < 0.001$
HPGD	0.774(0.684_0.877)	$p < 0.001$
ACSL1	0.691(0.601_0.795)	$p < 0.001$
SDHA	0.655(0.525_0.816)	$p < 0.001$
HMGCS1	0.676(0.567_0.806)	$p < 0.001$
HSD17B4	0.635(0.516_0.782)	$p < 0.001$
SLC22A5	0.705(0.602_0.824)	$p < 0.001$
LTC4S	0.409(0.179_0.932)	0.033
BPHL	0.569(0.465_0.695)	$p < 0.001$
ECI2	0.49(0.386_0.621)	$p < 0.001$
BCKDHB	0.705(0.584_0.852)	$p < 0.001$
SERINC1	0.686(0.598_0.787)	$p < 0.001$
INMT	0.793(0.702_0.896)	$p < 0.001$

BLVRA	0.742(0.582_0.945)	0.016
CD36	0.841(0.761_0.93)	$p < 0.001$
DLD	0.653(0.537_0.793)	$p < 0.001$
HCCS	0.599(0.459_0.782)	$p < 0.001$
MAOA	0.832(0.75_0.922)	$p < 0.001$
CA2	0.72(0.635_0.816)	$p < 0.001$
NBN	0.784(0.652_0.943)	0.01
AQP7	0.816(0.717_0.93)	0.002
ALDH1A1	0.791(0.72_0.87)	$p < 0.001$
AUH	0.56(0.47_0.668)	$p < 0.001$
HSDL2	0.548(0.449_0.668)	$p < 0.001$
ALAD	0.582(0.479_0.707)	$p < 0.001$
CEL	1.217(1.05_1.412)	0.009
LDHA	0.828(0.696_0.985)	0.034
CPT1A	0.621(0.525_0.734)	$p < 0.001$
SDHD	0.636(0.525_0.77)	$p < 0.001$
IDI1	0.597(0.487_0.733)	$p < 0.001$
GAD2	2.987(1.367_6.526)	0.006
ACSL5	0.829(0.708_0.97)	0.02
ECHS1	0.702(0.54_0.912)	0.008
ADIPOR2	0.727(0.555_0.954)	0.021
ENO2	1.182(1.027_1.361)	0.019
GABARAPL1	0.754(0.622_0.913)	0.004
ACADS	0.699(0.582_0.84)	$p < 0.001$
SUCLA2	0.6(0.511_0.705)	$p < 0.001$
APEX1	0.673(0.527_0.858)	0.001
PSME1	0.566(0.408_0.783)	$p < 0.001$
RDH11	0.642(0.503_0.818)	$p < 0.001$
ACOT2	0.733(0.59_0.91)	0.005
DLST	0.641(0.516_0.796)	$p < 0.001$
ACSM3	0.749(0.641_0.875)	$p < 0.001$
MLYCD	0.639(0.518_0.789)	$p < 0.001$
ENO3	1.356(1.164_1.579)	$p < 0.001$
ALDH3A2	0.618(0.536_0.713)	$p < 0.001$
CA4	0.784(0.713_0.862)	$p < 0.001$
ACOX1	0.526(0.41_0.675)	$p < 0.001$
ACAA2	0.615(0.534_0.707)	$p < 0.001$
IDH3B	0.535(0.382_0.751)	$p < 0.001$
ACSS1	0.744(0.609_0.909)	0.004
TP53INP2	0.521(0.424_0.64)	$p < 0.001$
GCDH	0.605(0.437_0.838)	0.003
ECH1	0.782(0.65_0.941)	0.009
YWHAH	0.698(0.544_0.897)	0.005

LGALS1	1.476(1.242_1.754)	$p < 0.001$
ACO2	0.665(0.581_0.762)	$p < 0.001$
PPARA	0.6(0.494_0.73)	$p < 0.001$
CBR1	0.723(0.55_0.95)	0.02
CBR3	1.198(1.014_1.417)	0.034

Abbreviations: OS: Overall survival; TCGA: The Cancer Genome Atlas; CI: Confidence interval.

p values less than 0.05 were considered to be statistically significant.
