# Supplementary figures and images for: A Potential Fatty Acid Metabolism-Related Gene Signature for Prognosis in Clear Cell Renal Cell Carcinoma
Source: Cancers (Basel). 2022 Oct 9;14(19):4943. doi: 10.3390/cancers14194943 (PMC9564311; doi:10.3390/cancers14194943)

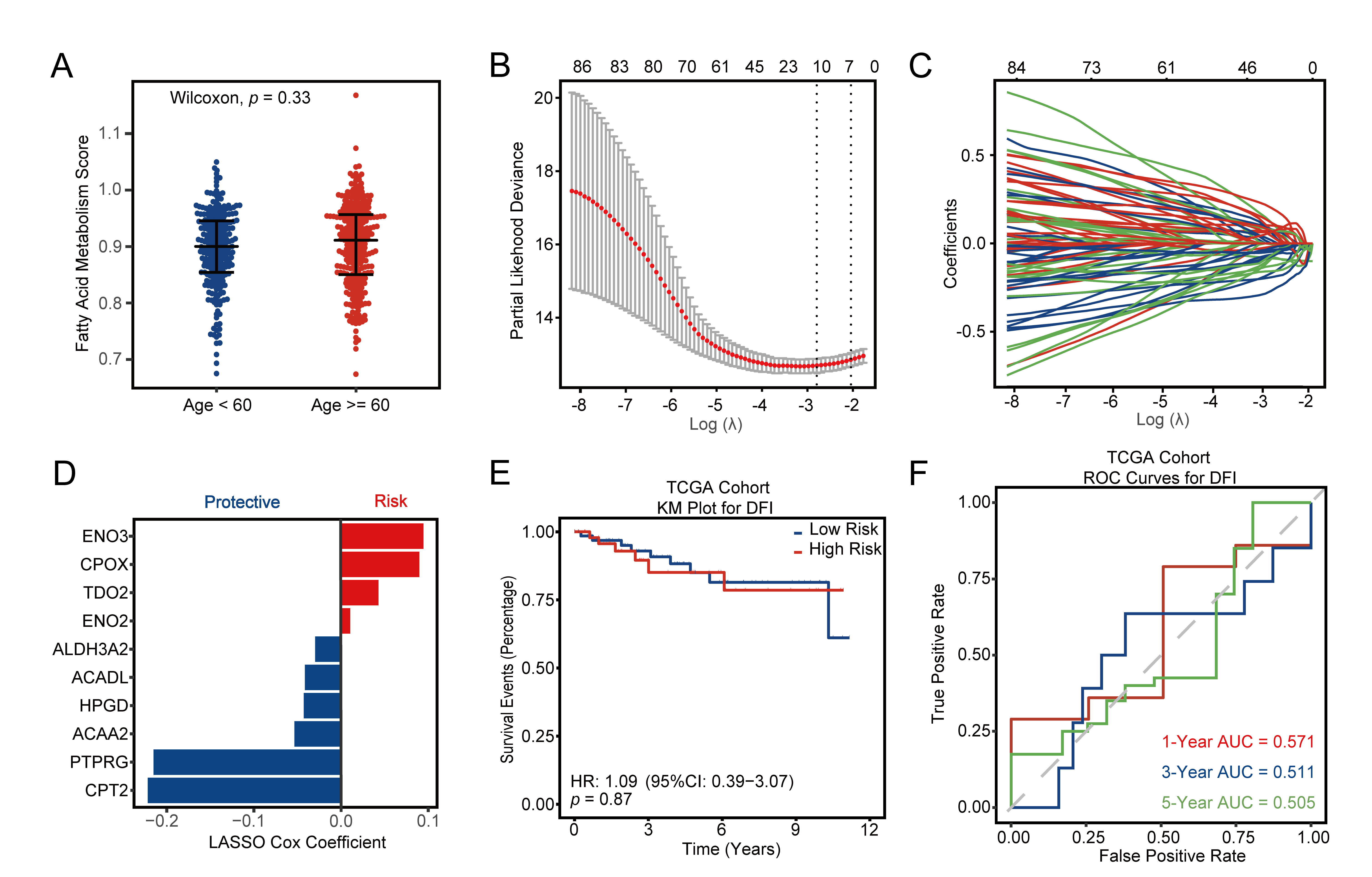

Supplement: Supplementary file 1 [file cancers-14-04943-s001.zip › Figure S1.tif]

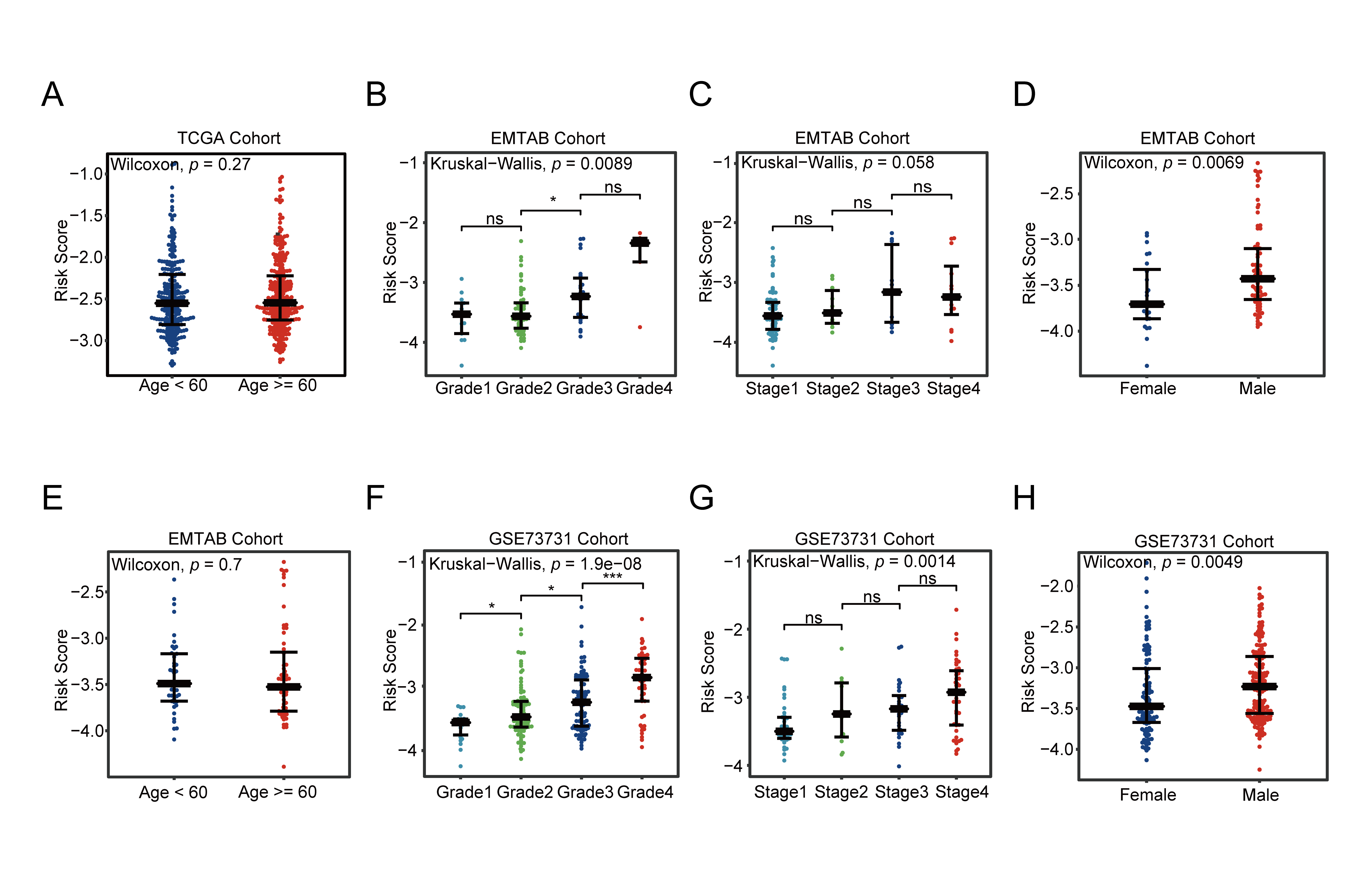

Supplement: Supplementary file 1 [file cancers-14-04943-s001.zip › Figure S2.tif]

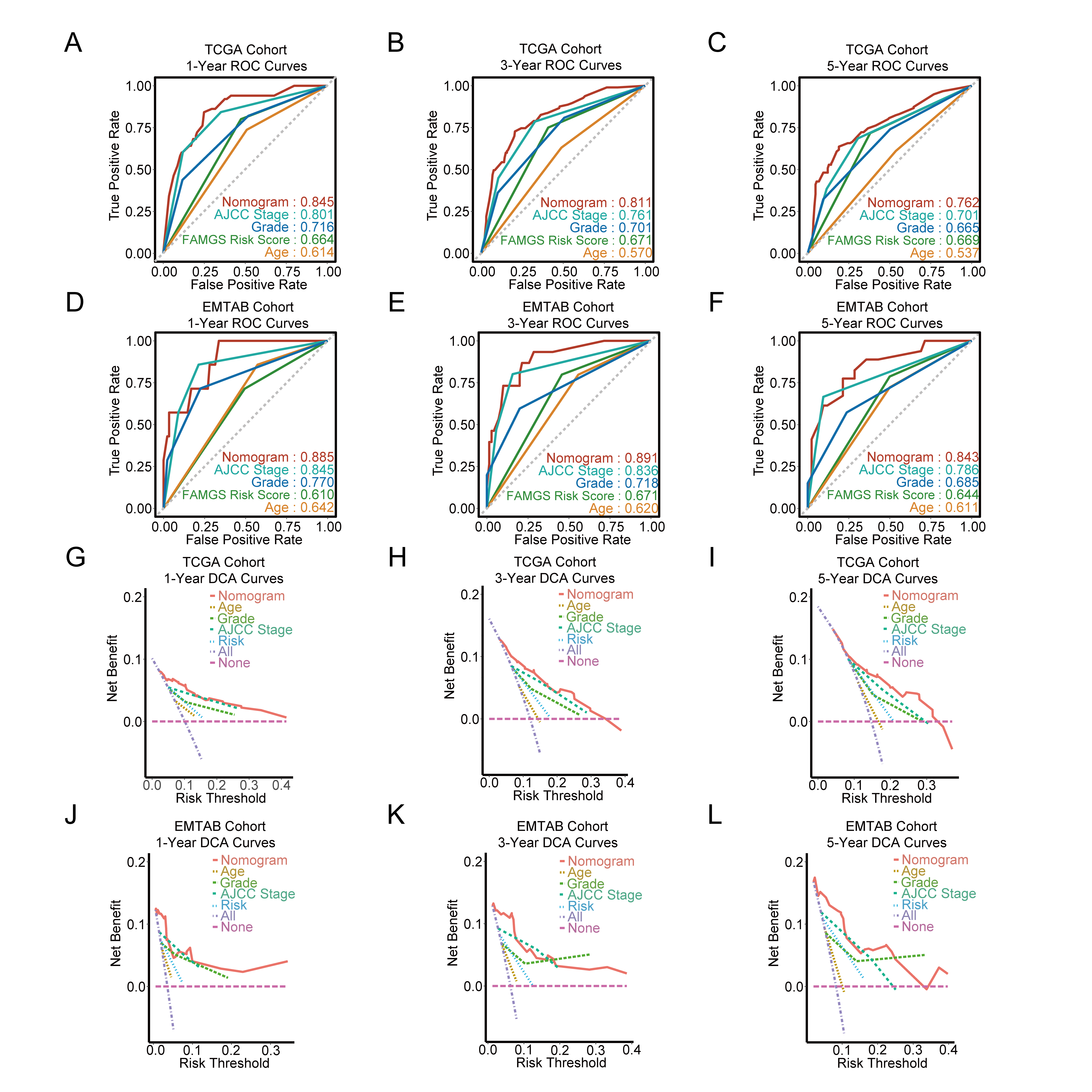

Supplement: Supplementary file 1 [file cancers-14-04943-s001.zip › Figure S3.tif]
